# mARC preoperative rectal cancer treatments vs. 3D conformal radiotherapy. A dose distribution comparative study

**DOI:** 10.1371/journal.pone.0221262

**Published:** 2019-08-16

**Authors:** Rocío Bermúdez Luna, María Victoria de Torres Olombrada

**Affiliations:** 1 Medical Physics Department, Hospital Universitario de Fuenlabrada, Fuenlabrada, Madrid, Spain; 2 Radiation Oncology Department, Hospital Universitario de Fuenlabrada, Fuenlabrada, Madrid, Spain; University of Seville, SPAIN

## Abstract

**Purpose:**

mARC (modulated arc) is the arc therapy technique provided by Siemens. The present study analyses the dose distributions and treatment times corresponding to preoperative rectal cancer mARC treatments. The results are compared to those corresponding to 3D-CRT plans.

**Methods:**

The plans of 30 patients, each having one mARC and one 3D-CRT plan, were evaluated. Every plan was calculated on a sequential two-phase treatment scheme with prescription doses of 45 Gy in the initial phase and 5.4 Gy in the boost phase. Dosimetric parameters and mean DVHs corresponding to the PTVs and OARs were assessed for both techniques.

**Results:**

All mARC plans were considered valid for treatment and yielded a highly significant improvement in the CI over 3D-CRT plans (p <0.001). They also showed statistically significant advantage on the parameters D98%, D95% and D2% of the high dose PTV. Regarding the OARs, mARC plans showed reductions in the mean dose of 3.5 Gy in the bladder and greater than 4 Gy in the femoral heads. Considering the small bowel, the mARC plans resulted in a 2.7 Gy mean reduction in the mean dose and lower irradiated volumes over the entire dose range.

**Conclusions:**

Arc therapy plans with the mARC technique for preoperative rectal cancer treatment in a sequential two-phase treatment scheme provide important advantages in the PTVs and OARs. mARC plans show superior protection of the femoral heads, bladder and small bowel, similar to the results found with other more widespread arc therapy techniques.

## Introduction

Rectal cancer represents an important oncological health problem. It was estimated that in 2018 approximately 43030 new rectal cancer cases would be diagnosed in the USA and rectal cancer would be the third cause of death due to oncological disease in that country [1].

Surgery is the only curative treatment of rectal cancer. Total mesorectal excision is considered the gold standard for this pathology [2]. Preoperative chemoradiotherapy has been demonstrated to lead to lower toxicity and lower local relapse rates than postoperative chemoradiotherapy and is regarded as the standard therapeutic approach in most clinical practice guidelines on locally advanced non metastatic rectal cancer [3–5]. With respect to radiotherapy treatment, it is of great significance to count on optimization strategies that can treat the target volume and, at the same time, reduce as much as possible the dose reaching the organs at risk (OARs). This is especially important in the case of the small bowel, as one of the most important secondary effects associated with pelvic radiotherapy for rectal cancer is gastrointestinal toxicity [6].

Volumetric modulated arc therapy (VMAT) has enabled the delivery of highly efficient radiotherapy plans and yields superior coverage and isodose conformation to the target volumes [7]. It also improves the OAR sparing in a variety of treatment sites in contrast to 3D conformal radiotherapy (3D-CRT) [7]. In comparison with fixed field intensity modulated radiotherapy (IMRT) treatments, the most significant contributions of VMAT treatments are the reduction of monitor units (MUs) and treatment time [7].

VMAT plans are commonly delivered when treating areas involving the pelvic lymph nodes, such as rectal cancer radiotherapy treatments. Target volumes in this region are usually concave shaped. The OARs that are contained inside the concavity, as is the case of the small bowel and the bladder, cannot be optimally protected with 3D-CRT. VMAT plans in this region result in dose distributions with stronger conformation and enable a superior sparing of these OARs.

The mARC (modulated arc) technique is the VMAT technique implemented by Siemens. It is based on the delivery of small arcs, called arclets, with a fixed multileaf collimator (MLC) conformation, alternated with gaps in which no radiation is emitted while the MLC leaves move to set the next beam’s shape. The mARC technique provides clinically acceptable dose distributions in a variety of locations [8]. It enables the delivery of treatments with a high degree of conformity to the target volumes, comparable to the conformity corresponding to prostate [9, 10] and head and neck [11] fixed field IMRT. It also generates dose distributions with a similar or superior OAR sparing to the one obtained with fixed field IMRT in both locations [9–11].

During 2016, the mARC technique was implemented in our centre. Preoperative rectal cancer radiotherapy was included among the different radiotherapy treatments that started being delivered with mARC after the commissioning process and comparison of the resulting dose distributions to those corresponding to 3D-CRT and IMRT plans. The former technique employed in our department to deliver preoperative rectal cancer radiotherapy treatments was 3D-CRT.

The objective of this study was to perform an analysis of the dosimetric parameters and the dose volume histograms (DVH) corresponding to the target volumes and the OARs of 30 rectal cancer patients treated with the mARC technique. The results were compared to those belonging to the 3D-CRT technique. To the best of our knowledge, this is the first study to report on the comparison between 3D-CRT and mARC in preoperative rectal cancer radiotherapy treatments. Treatment time corresponding to both techniques was also assessed.

## Material and methods

### Patient sample

Dosimetric parameters and DVHs of 30 rectal cancer patients receiving mARC treatment between October 2016 and August 2017 were analysed. The median age was 63.5 years (range: 47–77 years). Every patient had been diagnosed with locally advanced rectal cancer and had been submitted to simultaneous preoperative chemotherapy (capecitabine) and radiotherapy. The characteristics of the studied sample are shown in Table 1.

**Table 1:** Patient sample characteristics.

Parameter		No.	%
Gender	Male	15	50
	Female	15	50
T category	T2	2	6.7
	T3	21	70
	T4	7	23.3
N category	N0	3	10
	N1	13	43.3
	N2	12	40
	N+	2	6.7
M category	M0	28	93.3
	M1	1	3.3
	Mx	1	3.3
IUCC* stage	II	3	10
	III	26	86.7
	IV	1	3.3
Location	Low	3	10
	Middle	12	40
	High	11	36.7
	Datum not available	4	13.3

*IUCC: Union for international cancer control

### CT simulation and structure delineation

Every patient underwent a simulation CT scan on a Somatom Sensation Open (Siemens Healthcare, Erlangen, Germany) scanner.

Patients were asked to achieve a comfortably full bladder before the planning CT and every treatment fraction in order to reduce the bladder and small bowel doses. Patients were immobilised in prone position with extrinsic compression by means of a belly board (CIVCO Medical Solutions, Orange City, Iowa, USA) to attempt to minimize the irradiated intestinal volume and to reduce the small bowel doses [12].

Regarding the target volumes’ delineation, the gross target volume (GTV) was defined as the macroscopic tumour that is detectable by imaging techniques, specifically CT and MRI, the perirectal nodal structures and the soft tissue lesions which were suspected of being malignant.

The clinical target volumes (CTV) were delineated following the guidelines by Myerson et al. [13] and Valentini et al. [14]: CTVpelvis included the mesorectum, the posterior pelvic wall and the inner iliac lymph nodes. The lower pelvis was included in the case of tumours at a distance smaller than 6 cm from the anal verge, or with an affected sphincter, or in the case of a patient’s surgery being performed by means of abdominoperineal amputation. External iliac chains were contoured only in the event of involvement of the pelvic organs such as uterus, bladder, vagina, prostate or urethra. The inguinal chains were taken into account only in case of involvement of the anal sphincter or inferior third of the vagina. CTVboost contained the GTV and added the mesorectal area adjacent to the tumour plus a 2–3 cm margin.

The planning target volumes (PTVs) PTVpelvis and PTVboost were generated adding an isotropic 1 cm margin to the CTVpelvis and the CTVboost.

The organs at risk that were contoured and evaluated were the femoral heads, the bladder and the small bowel. The small bowel was outlined to 1 cm above the PTVboost.

Table 2 contains information on the CTV and PTV volumes.

**Table 2:** CTV and PTV volumes.

Parameter	Median	Range
CTVpelvis volume (cc)	668.8	(423.4–847)
PTVpelvis volume (cc)	1173.1	(845.3–1505)
CTVboost volume (cc)	259.8	(81.4–744.2)
PTVboost volume (cc)	425.0	(188.6–946.5)

### Dose prescriptions and organ at risk constraints

The evaluated plans corresponded to rectal cancer radiotherapy plans delivered on a sequential two-phase treatment scheme. The dose prescribed to the PTVpelvis corresponding to the initial phase was 45 Gy in 25 fractions (1.8 Gy/fraction). The second phase, or boost phase, had a prescription dose of 5.4 Gy in 3 fractions (1.8 Gy/fraction), delivered only to the PTVboost, which received an overall dose of 50.4 Gy.

The dosimetric requirement for each PTV was that at least 95% of its volume received 95% of the prescription dose with the OARs being irradiated with the lowest dose possible. The fulfilment of this condition was assessed on each plan phase and in the global plan, which comprised the sum of both phases. The overall results of the complete treatment and the OAR doses were evaluated on the global plan. The dose restrictions on the OARs are summarised in Table 3.

**Table 3:** OAR constraints for plan acceptability.

Organ at risk	Constraint
Bladder	V46 < 80%
Small bowel	V40 < 30%
	V40 < 150 cc
Femoral heads	V46 < 45%

### The mARC technique

The mARC technique is the VMAT technique provided by Siemens (Siemens Healthcare, Erlangen, Germany) and was implemented in our centre for the first time in our country in 2016. It is based on a “burst mode” delivery: the accelerator’s gantry rotates continuously around the patient while small arcs (called arclets) with dose delivery and defined MLC shape are alternated with gaps (called silent periods) in which no radiation is emitted and the MLC adapts its leaves to conform the shape of the next arclet’s beam. This contrasts the more widespread “conventional” VMAT techniques, in which radiation is emitted continuously while the MLC leaves move, the dose rate is adjusted and the gantry speed changes as it rotates around the patient [15–18].

Common arclet lengths vary from 2° to 5° [19]. Each arclet’s central point is known as the optimization point and it is where the treatment planning system (TPS) calculates the dose corresponding to the entire arclet. mARC’s dose delivery is the closest to the way the TPSs commonly calculate VMAT treatments: as a sum of static fields calculated on the gantry angles corresponding to the optimization points [8, 11, 19, 20]. During the treatment, the linear accelerator adjusts the gantry speed to deliver the planed MUs corresponding to each arclet. Further details on this irradiation technique can be found in the references [8, 11, 19, 20].

Radiotherapy treatments are delivered in our centre with two Siemens Artiste linear accelerators. Both of them are equipped with a 160 leaf MLC with 0.5 cm leaf width at the isocenter. The vast majority of mARC treatments are delivered with a flattening filter free (FFF) beam generated with an accelerating potential of 7 MV. This beam yields a dose rate of 2000 MU/min in beams with a corresponding number of MUs equal to or higher than 10. The depth corresponding to the maximum in the percentage depth dose is at 1.9 cm for a 10 cm x 10 cm field and 100 cm source to surface distance.

### Plan calculation

A mARC plan and a 3D-CRT plan were calculated for each patient, being the mARC plan the one considered for treatment. Every plan was calculated with the Eclipse version 13.6 TPS (Varian Medical Systems, Palo Alto, California, USA), the anisotropic analytical algorithm and a 2.5 mm dose calculation grid.

mARC plans were delivered with complete 360° arcs. An arclet size of 4° was selected on every calculation. This automatically generated 45 optimization points per arc as a consequence of how the Eclipse TPS calculates mARC treatments. Every plan was initially calculated with a single-arc and 30° collimator rotation in order to reduce the “tongue and groove” effect. In case of not fulfilling the requirements for plan acceptability on the PTVs and/or on the OARs, another complete arc was added to the plan. The second arc had a 330° collimator rotation. The 7 MV FFF previously mentioned beam was selected in every plan. Plans were optimised to spare the small bowel and the bladder as much as possible without losing PTV coverage. The inverse optimization process was first focused on achieving the criteria on the PTVs. Once this condition was fulfilled, successive optimizations were carried out to keep the OARs within their dose restrictions and to further attempt to lower as much as possible the dose they received while maintaining the PTV coverage.

3D-CRT was the former technique employed in our centre for rectal cancer radiotherapy treatments. 3D-CRT dose distributions were calculated with a 6 MV beam and the class solution we worked with in our department. This class solution was based on a set of three fields: a posteroanterior field and two lateral fields, as the most straightforward means to reduce as much as possible the dose to the bladder and small bowel. The weight of the beams was distributed so as not to generate regions receiving 95% of the prescription dose or higher doses outside the PTV’s immediate surroundings. The field-in-field technique was employed to reduce the PTV volume receiving more than 107% of the prescription dose as much as possible. If the coverage requirement on the PTV was not fulfilled, an anteroposterior field was added, and its weight was the minimum required to achieve it.

The calculation of a 3D-CRT plan in addition to the mARC plan did not interfere with the intended treatment and no decision regarding the patients’ treatment was made based on it. Every patient received the mARC treatment as planned and established in our centre after the mARC’s technical and dosimetric commissioning processes.

The study was approved by our centre’s research ethics committee (Comité Ético de Investigación del Hospital Universitario de Fuenlabrada / Fuenlabrada University Hospital’s Research Ethics Committee). The dosimetric data set corresponding to each patient’s DVHs was exported from the TPS and fully anonymised before its inclusion in the study. The study was completely performed with non-identifiable data and no further interaction with patients was required. Therefore, informed consent was not requested by the ethics committee.

### Evaluated parameters

The number of MU corresponding to the initial phase and the boost phase were studied for each technique.

Regarding the PTVs, the following values were characterised: homogeneity index (HI), conformity index (CI), near minimum dose (D98%), minimum dose covering the 95% of the PTV (D95%), median dose (D50%), near maximum dose (D2%), V95% and V107%.

The HI was calculated with the formula suggested by ICRU [21]:

$$HI=\frac{D2\%-D98\%}{D50\%}$$

The CI was calculated with the expression recommended by the RTOG [22]:

$$CI=\frac{V_{95\%isodose}}{V_{PTV}}$$

The OARs included in this study were the femoral heads, the bladder and the small bowel. Mean and maximum doses delivered to the OARs were studied, as well as the dosimetric parameters considered in the requirements for plan acceptability (Table 3).

Mean DVHs of the PTVs and OARs were also calculated for each sample of plans.

Finally, the treatment time corresponding to both techniques was analysed. Treatment time was considered as the time elapsed from the start of the irradiation to the completion of the treatment fraction, without taking into account the time spent on loading the plan and on image guidance procedures.

### Statistical analyses

Statistical tests were conducted on the evaluated parameters to find out whether statistically significant differences between both techniques exist. The normality of the distributions was verified through the Shapiro-Wilk test. To assess the variance homogeneity, Levene’s test was performed. If the data set corresponding to a particular parameter fulfilled the conditions of being normally distributed and showing variance homogeneity, the paired Student’s t-test was performed. Otherwise, the Wilcoxon signed rank test was conducted. The statistical tests were performed with SPSS version 15.0 (Statistical Package for Social Sciences, Chicago, Illinois, USA) software. p values equal or lower than 0.05 were considered statistically significant.

## Results

Every mARC plan, including the individual phase plans and the global plans, was evaluated by a radiation oncologist and was considered valid for treatment.

Two arcs were needed to meet the dosimetric requirements on the PTVs and the organs at risk in 11 initial phase plans and in one boost phase plan. In the rest of the plans, the dosimetric goals were reached with a single-arc.

### Target volumes

Distinction was made between the global plan comprising the sum of both phases and the results corresponding to each phase. Table 4 shows the results of the evaluated dosimetric parameters according to the delivery technique. PTVboost_sum and PTVpelvis_sum are the same as PTVboost and PTVpelvis. The expression “_sum” specifies that the associated evaluated items HI, CI, D98%, D95%, D50% and D2% of each of them correspond to the sum plan, and not to the individual phase plans. The items of the individual phase plans are the ones related to PTVpelvis and PTVboost.

**Table 4:** Results of the statistical analyses of the dosimetric parameters corresponding to the PTVs.

	Plan parameter	3D-CRT	mARC	p
PTVboost_sum	HI	0.09 ± 0.02	0.07 ± 0.02	0.001
	CI	2.84 ± 0.52	1.86 ± 0.35	< 0.001
	D98% (Gy)	48.4 ± 0.9	49.0 ± 0.8	0.001
	D95% (Gy)	48.9 ± 0.7	49.6 ± 0.6	< 0.001
	D50% (Gy)	51.1 ± 0.4	51.0 ± 0.2	n.s.
	D2% (Gy)	52.8 ± 0.4	52.4 ± 0.5	0.009
PTVpelvis_sum	HI	0.17 ± 0.03	0.17 ± 0.02	n.s.
	CI	1.84 ± 0.15	1.18 ± 0.08	< 0.001
	D98% (Gy)	44.1 ± 1.5	43.8 ± 0.8	n.s.
	D95% (Gy)	45.1 ± 1.5	45.0 ± 0.8	n.s.
	D50% (Gy)	49.8 ± 1.1	49.2 ± 1.2	< 0.001
	D2% (Gy)	52.6 ± 0.4	52.1 ± 0.5	< 0.001
PTVpelvis	MU	274.4 ± 12.7	682.7 ± 82.1	< 0.001
	HI	0.10 ± 0.01	0.11 ± 0.01	0.015
	CI	1.56 ± 0.15	1.03 ± 0.08	< 0.001
	D98% (Gy)	42.5 ± 0.5	42.1 ± 0.3	0.001
	D95% (Gy)	43.2 ± 0.4	43.0 ± 0.2	0.028
	D50% (Gy)	45.5 ± 0.2	45.5 ± 0.2	n.s.
	D2% (Gy)	47.2 ± 0.4	47.2 ± 0.3	n.s.
PTVboost	MU	283.8 ± 32.5	758.4 ± 108.3	< 0.001
	HI	0.10 ± 0.01	0.11 ± 0.02	n.s.
	CI	1.61 ± 0.12	1.01 ± 0.02	< 0.001
	D98% (Gy)	5.11 ± 0.04	5.09 ± 0.06	n.s.
	D95% (Gy)	5.19 ± 0.05	5.19 ± 0.04	n.s.
	D50% (Gy)	5.51 ± 0.04	5.47 ± 0.04	0.004
	D2% (Gy)	5.69 ± 0.04	5.66 ± 0.06	n.s.

The listed data are the mean value ± standard deviation; n.s.: not significant.

The number of MU increased considerably in the arc therapy plans due to the segmented irradiation that characterises this type of delivery.

**Fig 1:**
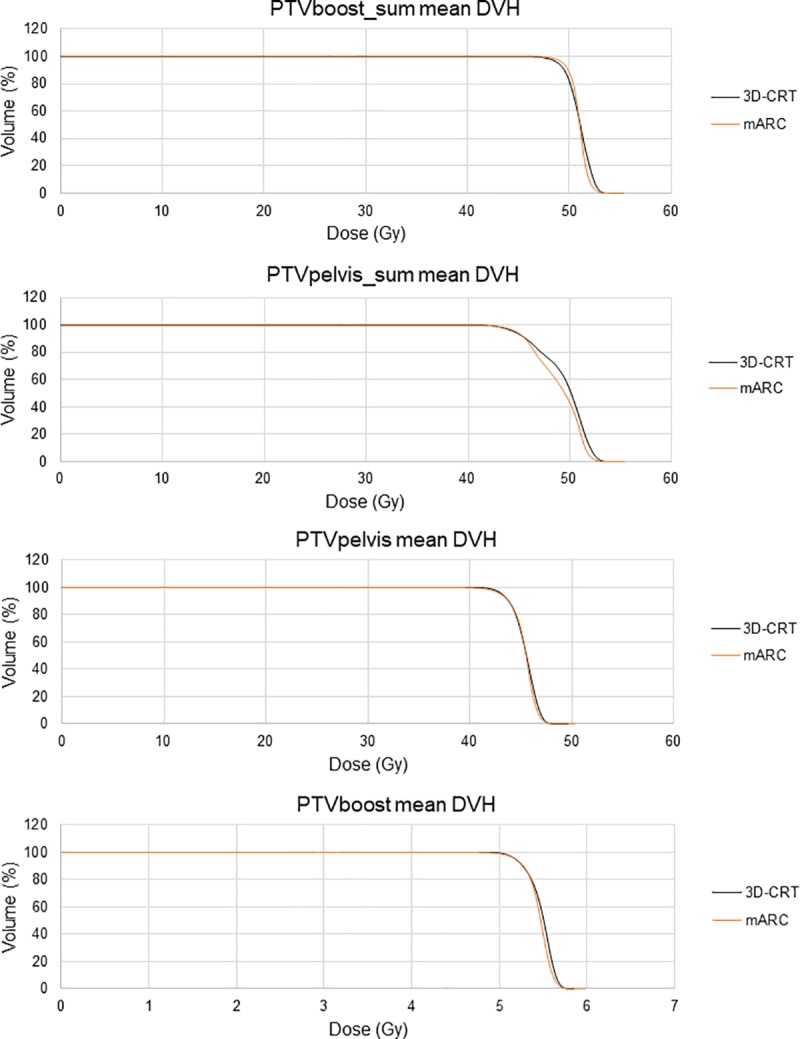
Mean dose volume histograms of the target volumes corresponding to each delivery technique.

The results corresponding to the mARC plans show a highly significant improvement in the CI over 3D-CRT plans. The HI improved significantly in the PTVboost_sum. The parameters D98% and D95% were significantly better in the PTVboost_sum, showing mean differences of 0.6 Gy and 0.7 Gy respectively when compared to the 3D-CRT plans. mARC plans yielded a significant reduction in the D2% in both PTVs when considering the global plans.

(Fig 1) shows the mean DVHs of the PTVs for each delivery technique.

Table 5 provides support to what is graphically displayed in (Fig 1), specifying the V95% and V107% values. The most remarkable result is the reduction obtained with the mARC plans in the V107% of the PTVpelvis_sum: a highly significant decrease of 9.3%. V95% of the mARC plans showed a significant improvement of 0.9% in the PTVboost_sum and a reduction of 1% in the PTVpelvis.

**Table 5:** Results of the statistical analyses of the V95% and V107% parameters.

	Plan parameter	3D-CRT	mARC	p
PTVboost_sum	V95% (%)	98.4 ± 1.7	99.3 ± 0.8	0.045
	V107% (%)	0.03 ± 0.11	0.03 ± 0.10	n.s.
PTVpelvis_sum	V95% (%)	99.0 ± 1.3	99.1 ± 0.6	n.s.
	V107% (%)	73.6 ± 15.7	64.3 ± 16.5	< 0.001
PTVpelvis	V95% (%)	97.0 ± 1.7	96.0 ± 0.8	0.041
	V107% (%)	0.08 ± 0.26	0.11 ± 0.19	n.s.
PTVboost	V95% (%)	97.2 ± 1.2	97.0 ± 1.1	n.s
	V107% (%)	0.11 ± 0.28	0.18 ± 0.35	0.033

The summarised data are the mean value ± standard deviation; n.s.: not significant.

### Organs at risk

**Fig 2:**
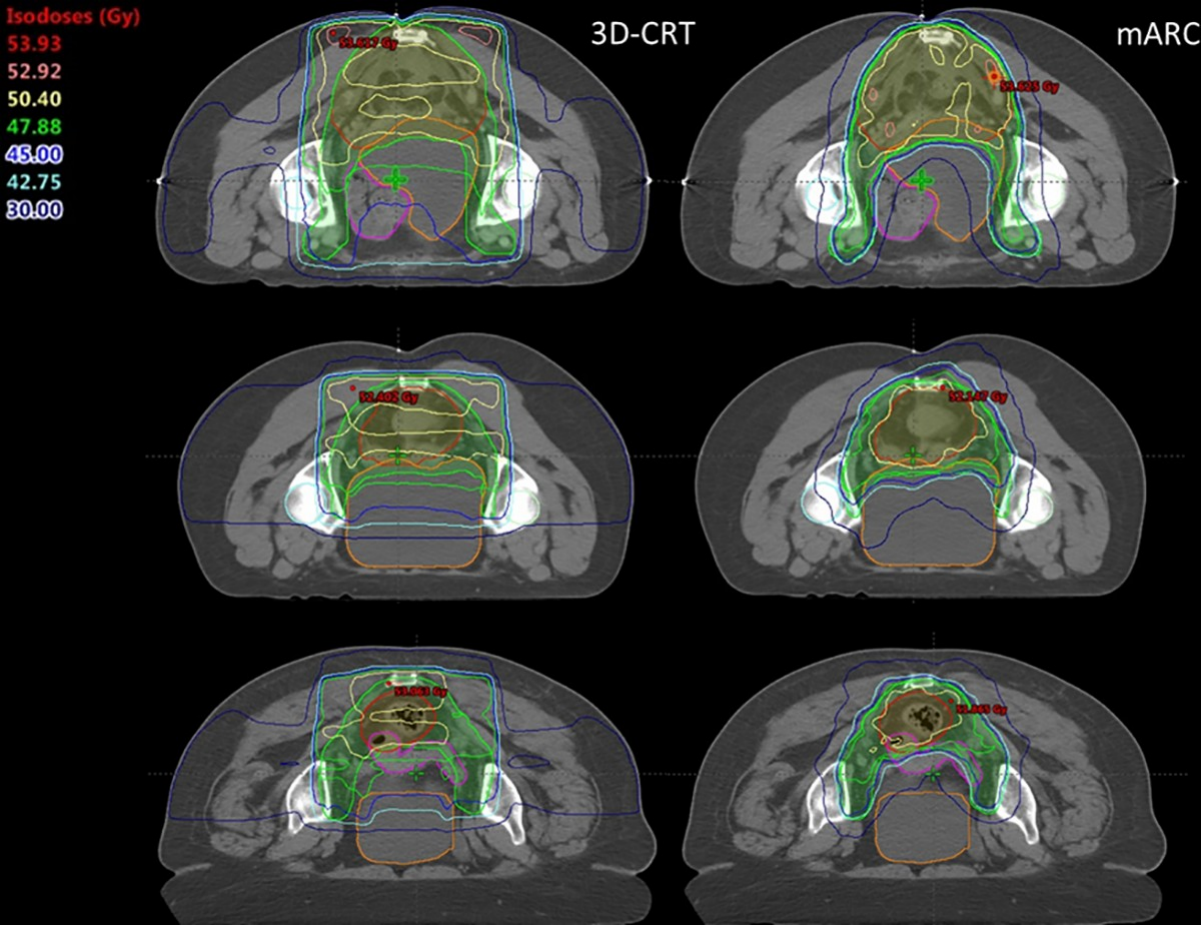
Dose distributions on an axial plane of three of the patients included in this study. The following structures are displayed: PTVpelvis in green, PTVboost in red, right femoral head in light green, left femoral head in light blue, bladder in orange, small bowel in pink.

The evaluated dosimetric parameters of the OARs correspond to the results of the complete treatment that includes the sum of both phases.

(Fig 2) shows images containing the dose distributions on an axial plane of three of the patients included in the present study. All of them correspond to sum plans. The different images contain the OARs that were evaluated in this study. The superiority of the mARC plans on the protection of the OARs can be observed.

Table 6 summarises the statistical analyses regarding the dosimetric parameters of the OARs.

**Table 6:** Results of the statistical analyses concerning the dosimetric parameters of the OARs.

	Plan parameter	3D-CRT	mARC	p
Bladder	Dmean (Gy)	36.4 ± 4.7	32.9 ± 4.8	< 0.001
	Dmax (Gy)	51.1 ± 1.6	51.8 ± 1.8	0.007
	V46 (%)	27.7 ± 15.8	14.0 ± 9.7	< 0.001
Small bowel	Dmean (Gy)	19.1 ± 10.3	16.4 ± 9.3	< 0.001
	Dmax (Gy)	50.3 ± 2.6	50.3 ± 3.5	n.s.
	V40 (%)	20.0 ± 16.8	13.8 ± 12.7	< 0.001
	V40 (cc)	73.8 ± 75.6	49.9 ± 52.7	< 0.001
Right femoral head	Dmean (Gy)	18.1 ± 6.3	13.7 ± 3.9	< 0.001
	Dmax (Gy)	46.0 ± 3.1	37.8 ± 4.1	< 0.001
	V46 (%)	0.6 ± 1.5	0.0 ± 0.0	< 0.001
Left femoral head	Dmean (Gy)	19.2 ± 6.4	14.4 ± 4.4	< 0.001
	Dmax (Gy)	45.9 ± 3.1	37.8 ± 4.8	< 0.001
	V46 (%)	0.4 ± 0.9	0.0 ± 0.0	0.001

**Fig 3:**
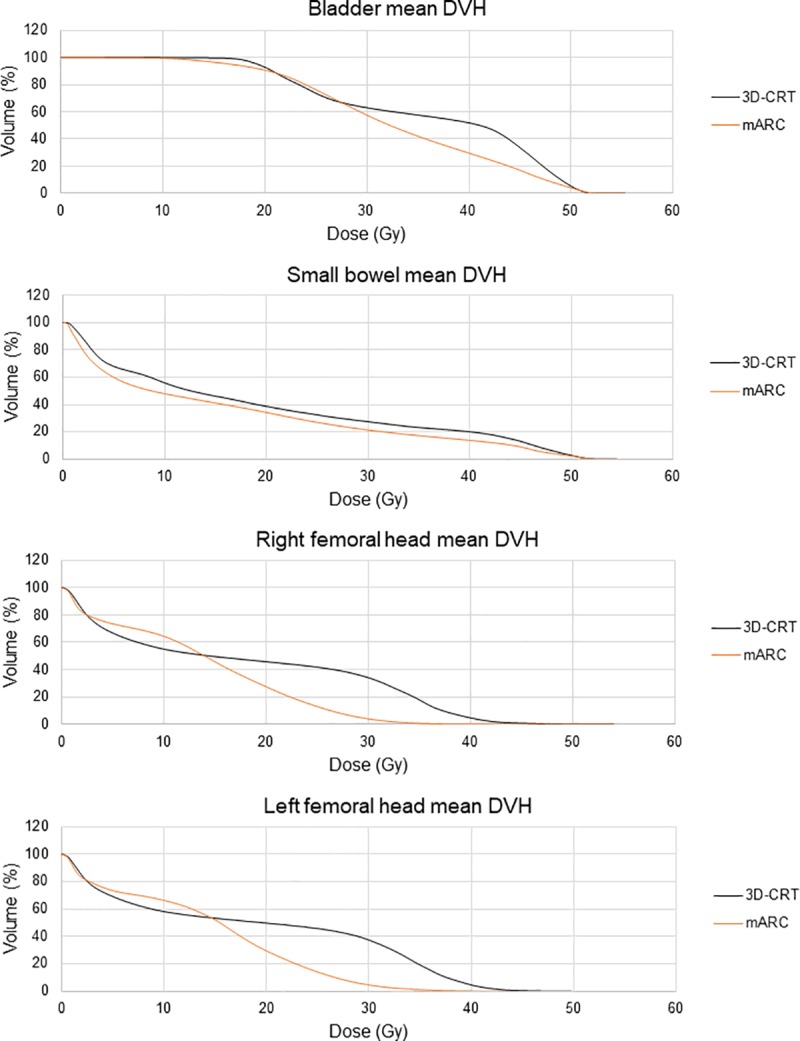
Mean dose volume histograms of the OARs corresponding to each delivery technique.

The listed data are the mean value ± standard deviation; n.s.: not significant.

(Fig 3) shows the mean DVHs corresponding to each technique.

With regard to the OARs, all the evaluated dosimetric parameters, except for the maximum small bowel dose, showed statistically significant differences between both techniques. Regarding the bladder, mARC plans showed a mean reduction of 3.5 Gy in the mean dose and 13.7% in the V46 parameter in comparison with 3D-CRT plans. As it is displayed in the mean DVH, the mARC dose distributions showed an advantage in the protection of the bladder volume receiving doses higher than 27 Gy. Considering the mARC results, mARC plans yielded a 2.7 Gy mean reduction in the small bowel’s mean dose. The V40 parameter showed a mean reduction of 6.2% and 23.9 cc. Mean DVHs showed a decrease of the small bowel irradiated volumes over the entire dose range. The femoral heads are the organs that showed the highest dose reduction when comparing 3D-CRT plans with mARC plans. Mean reductions in the mean dose of 4.4 Gy in the right femoral head and of 4.8 Gy in the left femoral head were obtained. mARC plans yielded a mean reduction of approximately 8 Gy in the maximum dose. Mean DVHs showed the sparing superiority of the mARC technique in the femoral head volume receiving dose values over 14 Gy.

### Treatment time

Table 7 contains the mean treatment time corresponding to 3D-CRT plans and mARC plans.

**Table 7:** Mean treatment time of the initial phase according to technique.

	Treatment phase	3D-CRT	mARC (1 arc)	mARC (2 arcs)
Treatment time (min:sec)	Initial phase	3:36	3:09	6:00

## Discussion

The present study shows the dosimetric results of the first preoperative rectal cancer treatments delivered in our centre using the mARC technique. To the best of our knowledge, this is the first study reporting on rectal cancer dose distributions with this technique. It could be of interest to departments that are equipped with Siemens’ linear accelerators and have performed, or are planning to perform the upgrade in order to have the ability to deliver mARC treatments. It may also prove to be an interesting contribution to the literature, with data corresponding to a less widespread arc therapy technique. The results concerning the dose distributions were compared with those corresponding to 3D-CRT plans, which was the technique previously employed in our centre to deliver rectal cancer radiotherapy treatments.

The mARC technique yielded superior dose distributions regarding conformity than the ones belonging to 3D-CRT. The greatest benefit on the PTVs was obtained in the global plans. These plans showed a statistically significant improvement on the parameters D98%, D95% and D2% of the boost’s planning target volume. They also yielded a highly significant reduction of 9.3% in the V107% parameter of the PTVpelvis, likely due to the fact that their multi-angle incidence favors the reduction of the high dose region.

With respect to the OARs, the mARC plans enable enhanced sparing of the femoral heads and the two organs at risk that are contained inside the concavity formed by the PTVpelvis: the bladder and the small bowel. Arc therapy plans show a reduction in the femoral heads mean dose greater than 4 Gy and a reduction of 3.5 Gy in the bladder mean dose. Regarding the OAR mean DVHs, the mARC technique yields enhanced sparing in volumes receiving doses over 14 Gy in the femoral heads and over 27 Gy in the bladder.

Gastrointestinal toxicity is one of the most important secondary effects associated with pelvic radiotherapy for rectal cancer [6]. According to our results, the mARC technique shows reductions of 6.2% and 23.9 cc in the small bowel’s V40 parameter. The risk of gastrointestinal toxicity is related to the total administered dose and the volume of intestinal loops irradiated with high dose levels [23]. Gallagher et al. suggest that the absolute volume of intestinal loops that receive a dose of 45 Gy or more is associated with a significant increment in late gastrointestinal toxicity [24].

Additionally, the mARC plans provide superior small bowel sparing over the entire dose range and a reduction of 2.7 Gy in the mean dose. In relation to this result, the research results provided by Tho et al. [25], Baglan et al. [26] and Robertson et al. [27] show a strong dose-volume correlation between the volume of irradiated small bowel and acute Grade 2+ [25] or Grade 3+ [26, 27] small bowel toxicity at all dose levels in preoperative chemoradiotherapy treatments with 3D-CRT.

Regarding late radiotherapy-induced toxicity with 3D-CRT, rates ranging from 2% to 9% on late small bowel obstruction or perforation have been registered after partial organ irradiation with radiotherapy treatments for rectal cancer with prescription doses around 50 Gy [28].

Treatment time of the 3D-CRT plans and the single-arc mARC plans are very similar and range between 3 to 4 minutes. Double-arc plans are delivered in 6 minutes.

### Previous research on the mARC technique

Several studies on the mARC technique and its corresponding dosimetric results have been published so far. Salter et al. [20] published a research study previous to its clinical implementation, using a prototype version of the Prowess TPS for the plan calculation. Their results confirmed that efficient treatments with excellent accuracy corresponding to different locations could be delivered with the mARC technique. Kainz et al. [8] also performed a study before the use of the mARC technique in clinical practice comparing plans corresponding to this delivery technique with tomotherapy and “conventional” volumetric modulated arc therapy. This study evaluated dosimetric results, delivered MUs and treatment time. Dosimetric verifications were also performed on phantoms. The authors concluded that the mARC is a feasible technique and enables the delivery of clinically acceptable plans with corresponding lower treatment times in the majority of the considered cases than those corresponding to tomotherapy plans or “conventional” volumetric modulated arc therapy. In 2013, Siemens published the White Paper on the mARC technique [19] after its implementation in the clinical practice.

Dzierma et al. [9] carried out the first systematic planning study comparing the mARC technique with the intensity modulated radiotherapy (IMRT) technique in prostate treatments. They calculated the plans retrospectively with the Prowess TPS. The authors concluded that, with the mARC technique, it is possible to deliver valid plans for treatment similar in quality to the IMRT plans. They reported a significant reduction in the scattered dose when using the FFF beam and decreased treatment times lowering from approximately 5 minutes for the IMRT plans with the flattened 6MV beam to approximately the half when delivering the mARC plans with the 7MV FFF beam.

Sarkar et al. [29] published the first results using the Eclipse TPS’s module for mARC plan calculation. They obtained clinically relevant plans for different sites that could be delivered with high accuracy and with corresponding treatment times up to three times shorter than the treatment times corresponding to equivalent IMRT plans.

Bell et al. [10] performed the first study that evaluated the systematic generation of mARC plans with the Eclipse TPS. They compared the dose distributions corresponding to mARC and IMRT plans using the flattened 6MV beam and the FFF 7MV beam in prostate treatments. The calculated plans resulted in similar quality regarding the PTV coverage, comparable protection of the rectum and posterior rectal wall and an improved sparing of the bladder in the volume irradiated under 75 Gy. Their results showed significant reductions in the scattered dose when irradiating with the 7 MV FFF beam, in contrast to the flattened 6 MV beam. The plans combining the mARC technique and the 7 MV FFF beam achieved a factor 3 reduction in treatment times, as compared to the IMRT plans using the flattened 6 MV beam.

In another research study Bell et al. [11] analysed the results corresponding to hypopharynx mARC plans and compared them to the corresponding results of IMRT plans. They also distinguished between the flattened 6MV beam and the FFF 7MV beam. They obtained plans of comparable quality in each modality. The parameters which showed statistically significant differences between both techniques and energies turned out to be in most cases more favourable to the mARC technique and the FFF 7MV beam, although the differences were small and were considered without clinical relevance. The authors observed a mean reduction in treatment times of more than 3 minutes with the mARC plans in combination with the FFF 7MV beam when compared to the IMRT plans that employed the 6MV beam.

### Previous studies on rectal cancer radiotherapy treatments with other arc therapy techniques

Several works have focused on the comparison of the dose distributions obtained with different arc therapy techniques with 3D-CRT dose distributions in rectal cancer radiotherapy treatments.

Duthoy et al. [30] published the first study on the dosimetric results of intensity modulated arc therapy for rectal cancer treatments. In this work, dosimetric aspects and viability of the arc therapy and 3D-CRT were compared. The dose prescribed to the target volume was 45 Gy in 25 fractions. Regarding the dosimetric results, the authors observed that the arc therapy plans yielded significant dose reductions in the bladder and the small bowel without compromising the dose to the PTV. Concerning the small bowel, the mean DVH showed that the arc therapy plans resulted in lower irradiated volumes over the entire dose range, as our results show. Longer treatment times were registered with the arc therapy plans, although they remained within a 5 to 10 minute time slot.

Richetti et al. [31] compared the results of 25 RapidArc and 20 3D-CRT rectal cancer plans corresponding to treated patients. The majority of the plans had a dose prescription of 44 Gy in 2 Gy fractions or 45 Gy in 1.8 Gy fractions. With respect to the PTV, they obtained a significant improvement in the conformity index with the RapidArc plans, comparable coverage and lower maximum doses. Regarding the femoral heads, they observed a significant mean reduction of 2.1 Gy in the mean dose with arc therapy. The mean DVHs showed the superiority of arc therapy plans in the protection of the volume being irradiated with doses over approximately 9 Gy. Considering the small bowel, the authors also observed with the RapidArc plans a decrease in the mean dose of 2.8 Gy and an enhanced sparing at all dose levels. Treatment times lowered from 3.4 minutes for the 3D-CRT plans to 2 minutes for the RapidArc plans.

Cilla et al. [32] studied the feasibility of volumetric modulated arc therapy with the Elekta Precise linear accelerator in rectal cancer treatments. The treatments were delivered with the simultaneous integrated boost technique, with prescription doses of 45 Gy and 57.5 Gy in 25 fractions. In order to perform a comparative analysis, an IMRT and a 3D-CRT plan were also calculated for every patient. Considering the comparison between arc therapy plans and 3D-CRT plans, both yielded similar PTV coverage. Arc therapy plans showed a significantly better conformity than 3D-CRT plans, although they yielded poorer homogeneity. With respect to the organs at risk, arc therapy plans showed a significantly superior sparing in volumes receiving doses higher than 15 Gy in the small bowel and higher than 30 Gy in the bladder. Arc therapy plans (all were 2-arc plans), as well as 3D-CRT plans were delivered in a mean treatment time of 5 minutes.

Wolff et al. [33] compared proton, RapidArc, IMRT and 3D-CRT preoperative rectal cancer treatment plans with prescription dose of 50.4 Gy in 28 fractions. This is the study with the closest prescription dose to ours, although without confining the last three fractions to a boost PTV. Regarding their results corresponding to the RapidArc and 3D-CRT plans, the conformity index to the PTV improved in the arc therapy plans. Arc therapy plans yielded a statistically significant reduction of 4.1 Gy in the bladder’s mean dose, close to our 3.5 Gy reduction, also showing statistically significant differences between 3D-CRT and arc therapy plans. The mean DVH showed the advantage of arc therapy plans in sparing of bladder volumes irradiated with doses higher than 30 Gy, which is a similar result to ours. Regarding the small bowel, the authors observed a statistically significant mean dose reduction of 2.9 Gy, similar to the value reported by Richetti et al. [31] and the one obtained in our study.

This last group published in a further study [34] the follow up results of patients who were treated with RapidArc plans and 3D-CRT plans, with the dose prescription mentioned in the previous paragraph [33]. They concluded that arc therapy plans yield the same tumour regression and reduce high grade acute and late toxicity.

## Conclusions

Arc therapy plans with the mARC technique for preoperative rectal cancer radiotherapy treatment in a sequential two-phase treatment scheme provide high quality dose distributions including important advantages in the PTVs and OARs. The superiority in isodose conformity enables improved sparing of the OARs while maintaining a good PTV coverage in comparison to the 3D-CRT technique.

In the global plan that comprises the sum of both phases and, regarding the high dose PTV, these plans show superior results in the HI, CI, D98%, D95% and D2% parameters. Statistically significant differences are observed in all these parameters when comparing between the mARC and the 3D-CRT techniques. mARC plans provide enhanced protection of the femoral heads, bladder and small bowel, showing statistically significant differences in the majority of the assessed parameters. Our results regarding the OARs are consistent with the findings provided by other studies that considered other more widespread arc therapy techniques: on one hand, a systematic sparing over the entire dose range and approximately 3 Gy mean dose reduction in the small bowel and, on the other hand, a dose reduction in the bladder, showing the mARC plans superior protection of the volume receiving doses higher than, approximately, 30 Gy or more.

Treatment times corresponding to single-arc treatments and 3D-CRT plans are very close, in the interval between 3 and 4 minutes.

Taking into account the results in the PTVs and OARs in the sequential two-phase treatments considered in this study, the mARC technique could be considered as a good basis to start delivering preoperative rectal cancer treatments with the simultaneous integrated boost technique.

## Supporting information

S1 Table Data regarding Table 2. (XLSX)

S2 Table Data regarding Tables 4, 5 and 6. (XLSX)

S3 Table Data regarding Table 7. (XLSX)

S4 Table Data corresponding to the depicted mean DVHs. (XLSX)
